# Integrative transcriptomic- neuroimaging analysis reveals polygenic correlates of interhemispheric functional decoupling in ischemic stroke

**DOI:** 10.3389/fncel.2026.1908231

**Published:** 2026-08-26

**Authors:** Ri-Bo Chen, Yu-Xuan He, Xin Huang, Chang Wang

**Affiliations:** 1 School of Medical Engineering, Henan Medical University, Xinxiang, Henan, China; 2 Department of Medical Imaging, Jiangxi Provincial People’s Hospital, The First Affiliated Hospital of Nanchang Medical College, Nanchang, Jiangxi, China; 3 School of Ophthalmology and Optometry, Jiangxi Medical College, Nanchang University, Nanchang, China; 4 The Affiliated Eye Hospital, Jiangxi Medical College, Nanchang University, Nanchang, China

**Keywords:** allen human brain atlas, functional homotopy, gene expression, resting-state functional MRI, stroke, voxel-mirrored homotopic connectivity

## Abstract

**Background:**

Functional magnetic resonance imaging (fMRI) has revealed abnormal brain activity patterns in stroke patients, yet the genetic correlates underlying functional homotopy – defined as synchronized spontaneous activity between bilateral homologous brain regions – remain poorly characterized. This study investigates the genetic basis of voxel-mirrored homotopic connectivity (VMHC) abnormalities in stroke patients.

**Methods:**

We analyzed resting-state fMRI data from 50 stroke patients and 50 healthy controls (HC) to quantify VMHC. Spatial transcriptome-neuroimaging correlations were established using the Allen Human Brain Atlas (AHBA) to identify VMHC-associated genes. Transcriptomic analyses combined pathway-centric functional annotation (DAVID) with protein-protein interaction (PPI) network modeling (STRING v12.0).

**Results:**

Stroke patients exhibited significantly reduced VMHC in the rectus gyrus, superior temporal gyrus, middle occipital gyrus, cuneus, and right calcarine/left posterior cingulate gyrus (*p* < 0.05, GRF-corrected). VMHC alterations correlated positively and negatively with 1,198 genes each. Transcriptomic profiling revealed significant enrichment in synaptic vesicle trafficking, mitochondrial energy metabolism, and neuroinflammation-related pathways. PPI mapping uncovered multi-tiered networks with hub genes including *BRCA1*, *CDK9*, *ACTB*, and *ATP6V1A/F* involved in transcriptional regulation, cytoskeletal dynamics, and vesicular acidification.

**Conclusion:**

This multimodal integration study elucidates polygenic correlates of post-stroke VMHC abnormalities, demonstrating that interhemispheric coordination may depend on synergistic interactions among functionally diverse gene clusters. Our findings provide a molecular framework for understanding post-stroke neural network reorganization and offer a link between functional neuroimaging phenotypes and gene expression, though all associations remain correlational and require mechanistic validation.

## Introduction

As evidenced by recent multinational studies, stroke stands as the third leading cause of combined disability and premature death worldwide (GBD 2017 Stroke Collaborators, 2018). Ischemic stroke, accounting for approximately 80% of cases, often involves the basal ganglia (Sacco et al., 2013; Araki et al., 1983). Following stroke, functional deficits – including visual, motor, sensory, and cognitive impairments – are linked to both the primary lesion and remote network disruptions (Kelly-Hayes et al., 2003; Grefkes and Fink, 2011). Over the past decades, significant advancements have been achieved in diagnosis and acute treatment, but the complex pathophysiological mechanisms underlying post-stroke brain network reorganization remain incompletely understood (Wu et al., 2019).

Resting-state functional magnetic resonance imaging (fMRI) has revealed that spontaneous brain activity is organized into large-scale networks, referred to as resting-state networks (Bullmore and Sporns, 2009; Fox et al., 2005). Functional homotopy – the high degree of synchronized spontaneous activity and functional co-activation between geometrically corresponding bilateral hemispheric regions – represents a fundamental aspect of the brain’s intrinsic functional architecture (Salvador et al., 2005; Stark et al., 2008). Voxel-mirrored homotopic connectivity (VMHC) provides a robust metric for assessing interhemispheric functional coordination by quantifying the temporal correlation of BOLD signals between mirrored voxel pairs (Zuo et al., 2010). Studies have shown that stroke induces alterations in multiple large-scale networks, including the default mode network and sensorimotor network, and disrupts interhemispheric functional homotopy (Ovadia-Caro et al., 2013; Wang et al., 2014). Such connectivity abnormalities are closely associated with motor, sensory, and cognitive impairments following stroke. Thus, investigating VMHC abnormalities provides an important window into post-stroke neurological dysfunction.

Although VMHC offers a valuable imaging phenotype, the underlying molecular correlates remain unclear. Transcriptome-imaging fusion analysis has emerged as a powerful approach to reveal the genetic basis of brain function abnormalities (Fornito et al., 2019). The Allen Human Brain Atlas (AHBA) provides spatially resolved gene expression profiles from six postmortem human brains, with over 20,000 genes mapped to 3,702 sampling sites, enabling direct correlation between molecular gradients and neuroimaging-derived phenotypes (Sunkin et al., 2013; Zhu et al., 2021). This paradigm has been applied across disorders: Prior integrative work has mapped functional connectivity phenotypes to transcriptional modules (Richiardi et al., 2015), and Buch et al., 2023 delineated ASD subtype-specific connectomic disruptions. Although stroke heritability studies implicate polygenic risk (Traylor et al., 2017), the molecular basis of post-stroke VMHC anomalies remains poorly understood.

**FIGURE 1:**
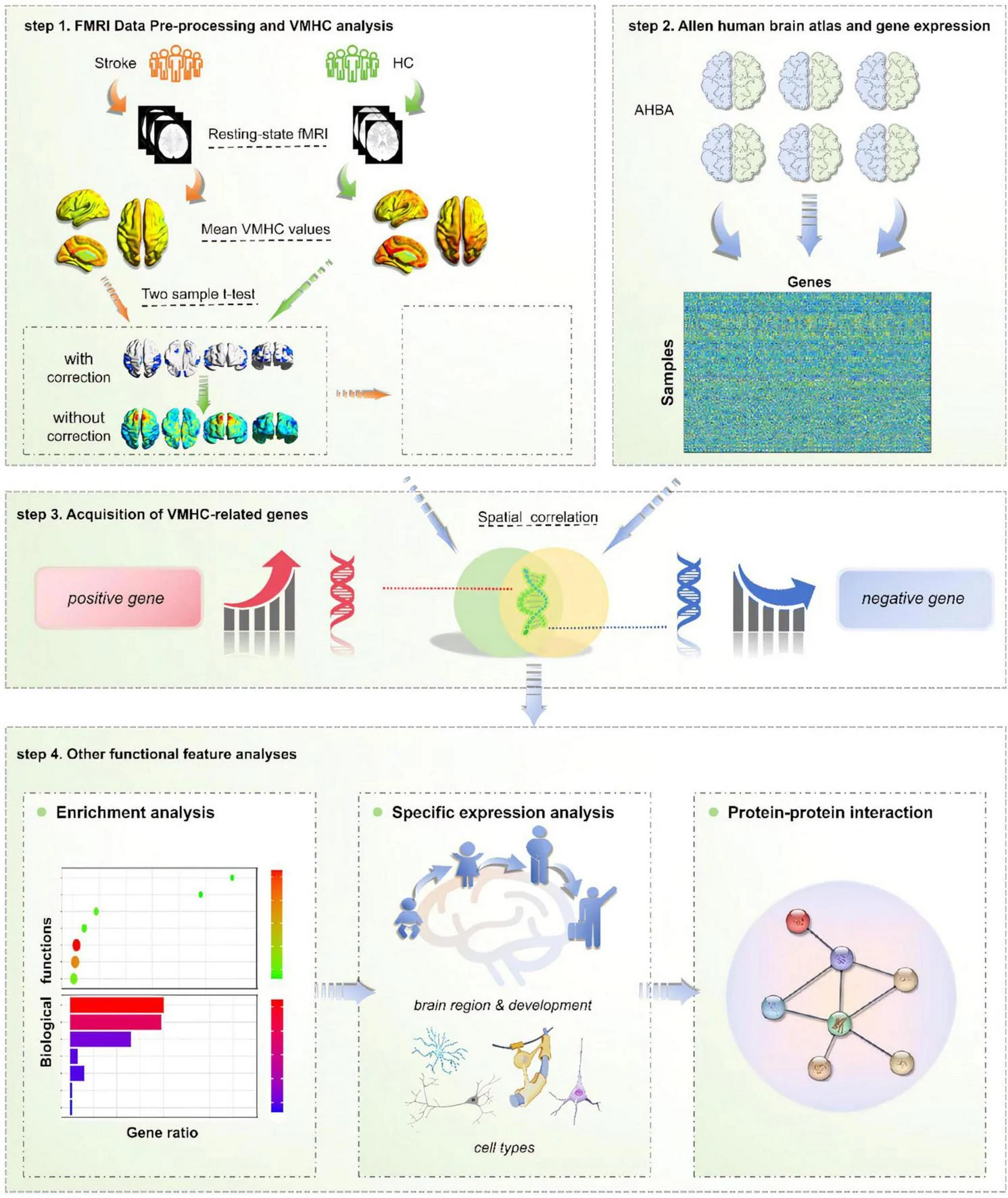
Study flowchart. Resting-state fMRI data from stroke patients and healthy controls were preprocessed to compute VMHC (Step 1). Gene expression matrices were obtained from the Allen Human Brain Atlas (Step 2). Transcriptome-neuroimaging association analysis using PLS regression identified VMHC-correlated genes (Step 3). Functional enrichment, PPI network, and spatiotemporal expression analyses were performed (Step 4).

In this study, we integrated neuroimaging and gene expression data to investigate the genetic correlates underlying stroke-related VMHC differences. Resting-state fMRI was collected from 50 stroke patients and 50 HC. VMHC values were computed and group differences evaluated. Using AHBA gene expression data, a transcriptome-neuroimaging association analysis was performed to identify genes correlated with VMHC alterations. Finally, functional enrichment, PPI, and spatiotemporal expression analyses were carried out to explore biological significance. The research process is illustrated in Figure 1.

## Materials and methods

### Participants

A total of 100 participants were enrolled from Jiangxi Provincial People’s Hospital, comprising 50 first-onset acute ischemic stroke patients and 50 HC matched for age, sex, and educational background. Stroke diagnosis adhered to the Chinese Guidelines for Acute Ischemic Stroke Management (2018), confirmed via multimodal MRI (including diffusion-weighted imaging) and neurological evaluations by two board-certified neurologists. All participants were right-handed (Edinburgh Handedness Inventory score > 40) with comparable demographics (mean age: 57.73 ± 17.21 years vs. 56.32 ± 16.63 years); male/female (ratio: 27/23 vs. 27/23). Demographic characteristics are detailed in Table 1.

**TABLE 1:** Demographic characteristics.

Condition	Stroke group	HC group	T/χ^2^-value	*p*-value	Effect size (Cohen’s d)	95% CI
Sex (male/female)	27/23	27/23	N/A	1	N/A	N/A
Age (years)	57.73 (17.21)	56.32 (16.63)	0.42	0.69	0.084	[−0.31, 0.48]
Education (years)	11.71 (2.32)	11.54 (2.17)	1.232	0.217	0.077	[−0.30, 0.45]

HC, healthy control; CI, confidence interval.

Inclusion criteria for patients: (1) lesion localization in the basal ganglia or semiovale region; (2) clinical symptoms (dizziness, hemiplegia, or aphasia) leading to MRI-confirmed acute stroke; (3) age 40–75 years. Exclusion criteria: (1) MRI contraindications (e.g., ferromagnetic implants, pacemakers); (2) history of neuropsychiatric disorders (e.g., Alzheimer’s disease, schizophrenia, epilepsy) or structural brain abnormalities (e.g., severe brain atrophy, prior stroke, tumors, trauma); (3) concurrent participation in interventional drug trials. HC had no cognitive impairment or major systemic comorbidities.

### MRI acquisition

All neuroimaging scans were performed on a 3T Discovery MR750W system (GE Healthcare) with an 8-channel phased-array head coil. Participants were stabilized with foam pads, and earplugs were provided for noise attenuation. During resting-state fMRI acquisition, participants kept their eyes closed and remained relaxed. Imaging parameters: (1) BOLD: 240 time points; TR/TE = 2000/25 ms; flip angle 90°; FOV = 240 × 240 mm^2^; matrix 64 × 64; 40 axial slices; slice thickness 3.5 mm (Zhang et al., 2018); (2) T1-weighted anatomical: TR/TE = 8.0/3.0 ms; flip angle 12°; FOV = 240 × 240 mm^2^; matrix 256 × 256; slice thickness 1.0 mm; voxel size 1 mm^3^ isotropic (Du et al., 2016).

### fMRI data preprocessing

Data were preprocessed using DPABI 8.0 (Yan et al., 2016). The pipeline included: DICOM-to-NIfTI conversion; removal of first 10 volumes; slice-timing correction; rigid-body motion realignment (translation < 1.5 mm, rotation < 1.5°); T1-guided nonlinear normalization to MNI152 space (3 × 3 × 3 mm^3^) with 6 mm FWHM Gaussian smoothing; linear detrending; and nuisance regression (Friston 24-parameter motion model, white matter, cerebrospinal fluid). Global signal regression was not performed, as this approach remains controversial in VMHC studies and may introduce anti-correlations that distort homotopic connectivity estimates (Murphy et al., 2009; Gotts et al., 2013). Scrubbing was not applied, as the proportion of volumes with framewise displacement (FD) >0.5 mm was less than 5% across all participants; FD time series were included as covariates.

### VMHC analysis

VMHC was calculated as the Pearson correlation between BOLD time series of mirrored interhemispheric voxel pairs, followed by Fisher’s z-transformation (Zuo et al., 2010). VMHC was computed only for voxels within the gray matter mask (probability > 0.2) to reduce white matter and CSF contamination. One-sample *t*-tests assessed within-group spatial patterns; two-sample *t*-tests compared stroke vs. control groups. Whole-brain analyses employed a two-stage correction: voxel-level *p* < 0.001 combined with Gaussian random field (GRF)-based cluster correction (*p* < 0.05). Significant clusters were visualized with BrainNet Viewer. Partial Pearson correlations were computed between regional zVMHC values and clinical metrics (NIHSS scores) in stroke patients, controlling for age and sex as covariates. Statistical significance was set at *p* < 0.05 (Bonferroni-corrected for multiple regional comparisons).

### Brain gene expression data processing

AHBA microarray data from six postmortem healthy brains (Sunkin et al., 2013) were processed using a standardized pipeline (abagen toolbox^1^; Markello et al., 2021). Probes with signal intensity below 50% of background noise were filtered. After filtering, 32,456 probes were retained from the original 58,692 probes. For each gene, the probe with the maximum expression value was selected, resulting in a final matrix of 3,702 sample points × 20,737 genes (probe selection retained 20,737 unique genes). Expression values were mapped to MNI coordinates. Due to the limited availability of right-hemisphere transcriptomic samples in the Allen Human Brain Atlas, our analyses were largely restricted to the left hemisphere.

### Gene expression-vMHC spatial correlation analysis

For each AHBA sampling point, the uncorrected whole-brain t-value from the stroke vs. control VMHC contrast was extracted as the response variable. We chose whole-brain t-values rather than restricting to significant clusters to maximize sensitivity to diffuse signals that may not survive stringent cluster-level correction but still carry biological relevance. This approach increases sensitivity at the cost of potential false positives; therefore, all identified gene associations are presented as exploratory findings requiring independent validation.

Partial least squares (PLS) regression was performed with gene expression as predictors (X) and *t*-values as response (Y), implemented in MATLAB PLS_Toolbox. PLS handles high-dimensional multicollinearity effectively. Spatial autocorrelation was addressed using a spatial smoothing approach with a Gaussian kernel (FWHM = 6 mm) applied to the gene expression data prior to PLS analysis, consistent with prior imaging-transcriptomic studies (Fornito et al., 2019). Additionally, we implemented a spin-test permutation procedure (1,000 permutations) to account for spatial autocorrelation in the gene-VMHC associations, with significance threshold set at *p* < 0.001. A total of 1,000 permutations were performed for the spin-test and PLS stability assessment, representing a pragmatic balance between computational feasibility and statistical precision, consistent with prior studies using similar sample sizes and spatial resolution (Richiardi et al., 2015). Ten-fold cross-validation was performed to assess model predictive performance (mean cross-validated predictive R^2^ = 0.38, SD = 0.05). The cumulative explained variance in the training model across the four retained components was 0.42. Permutation testing (1,000 permutations) confirmed that the observed PLS components explained significantly more variance than chance (*p* < 0.001). Genes with *p* < 0.001 were retained; the top 25% by absolute association strength were selected for downstream analyses. This threshold was chosen to balance statistical stringency with the retention of biologically relevant signals, as more stringent thresholds (e.g., top 10%) yielded insufficient genes for meaningful pathway enrichment (less than 500 genes per set). This approach is consistent with prior imaging-transcriptomic studies (Richiardi et al., 2015). These genes were divided into two sets: (1) positively correlated genes (higher gene expression associated with greater VMHC reduction, i.e., more negative *t*-values, *n* = 1,198) and (2) negatively correlated genes (higher gene expression associated with lesser VMHC reduction, i.e., less negative *t*-values, *n* = 1,198).

### Gene enrichment analysis

Functional enrichment profiling was conducted using DAVID v6.8 (Huang et al., 2009) for GO term enrichment (biological processes, cellular components, and molecular functions), and KEGG pathway analyses were performed separately (Kanehisa and Goto, 2000; Kanehisa et al., 2025). Enrichment results with Benjamini-Hochberg corrected *p* < 0.05 were visualized using the Weishengxin platform^2^. Bubble charts and bar charts represent the top seven GO terms and top 15 KEGG pathways.

### Protein-protein interaction analysis

PPI networks were constructed using STRING v12.0 (Szklarczyk et al., 2023) with high-confidence interactions (score ≥ 0.9). Hub genes were defined as the top 10% by node degree using CytoNCA in Cytoscape v3.9.1. Network topology metrics (average degree, clustering coefficient, network density) were calculated for each network. Spatiotemporal expression dynamics of hub genes were mapped using the Human Brain Transcriptome Atlas (Kang et al., 2011)^3^.

## Results

### Demographic and clinical characteristics

As shown in Table 1, there were no significant differences in sex, age, or education between groups (all *p* > 0.05), indicating good matching.

### Lesion characteristics

All patients presented with acute infarcts confined to the basal ganglia or semiovale center. Lesion side distribution was as follows: left hemisphere in 29 patients (58%) and right hemisphere in 21 patients (42%). Mean infarct volume was 3.8 ± 2.1 cm^3^ (range: 0.9–8.2 cm^3^), as measured on DWI sequences using semi-automated segmentation. These regions are primarily supplied by the lenticulostriate perforating arteries arising from the middle cerebral artery, resulting in a relatively homogeneous vascular territory profile across patients.

### Comparison of VMHC between stroke patients and HC

**FIGURE 2:**
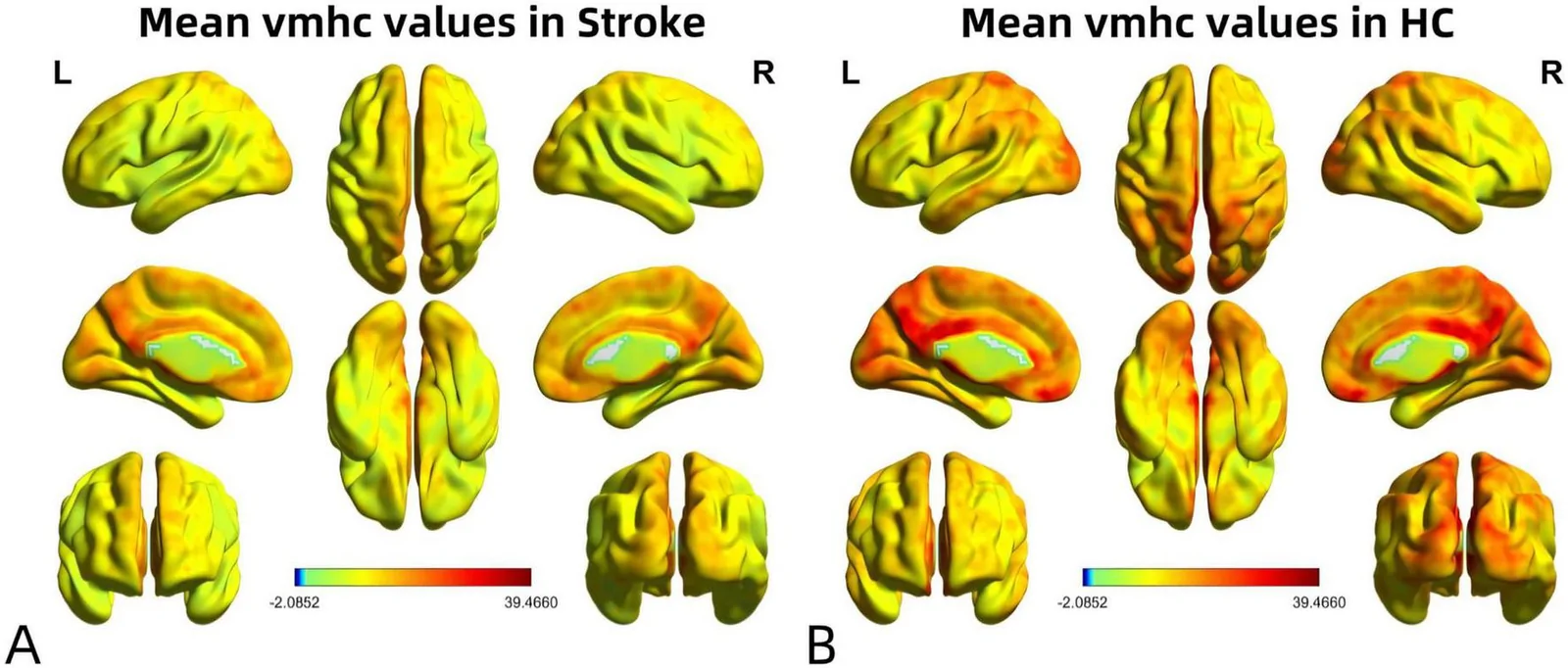
One-sample *t*-test VMHC maps for stroke and control groups. Spatial distribution of group mean zVMHC values in **(A)** stroke patients and **(B)** healthy controls (HC). Color bar represents *t*-values. L, left; R, right.

**FIGURE 3:**
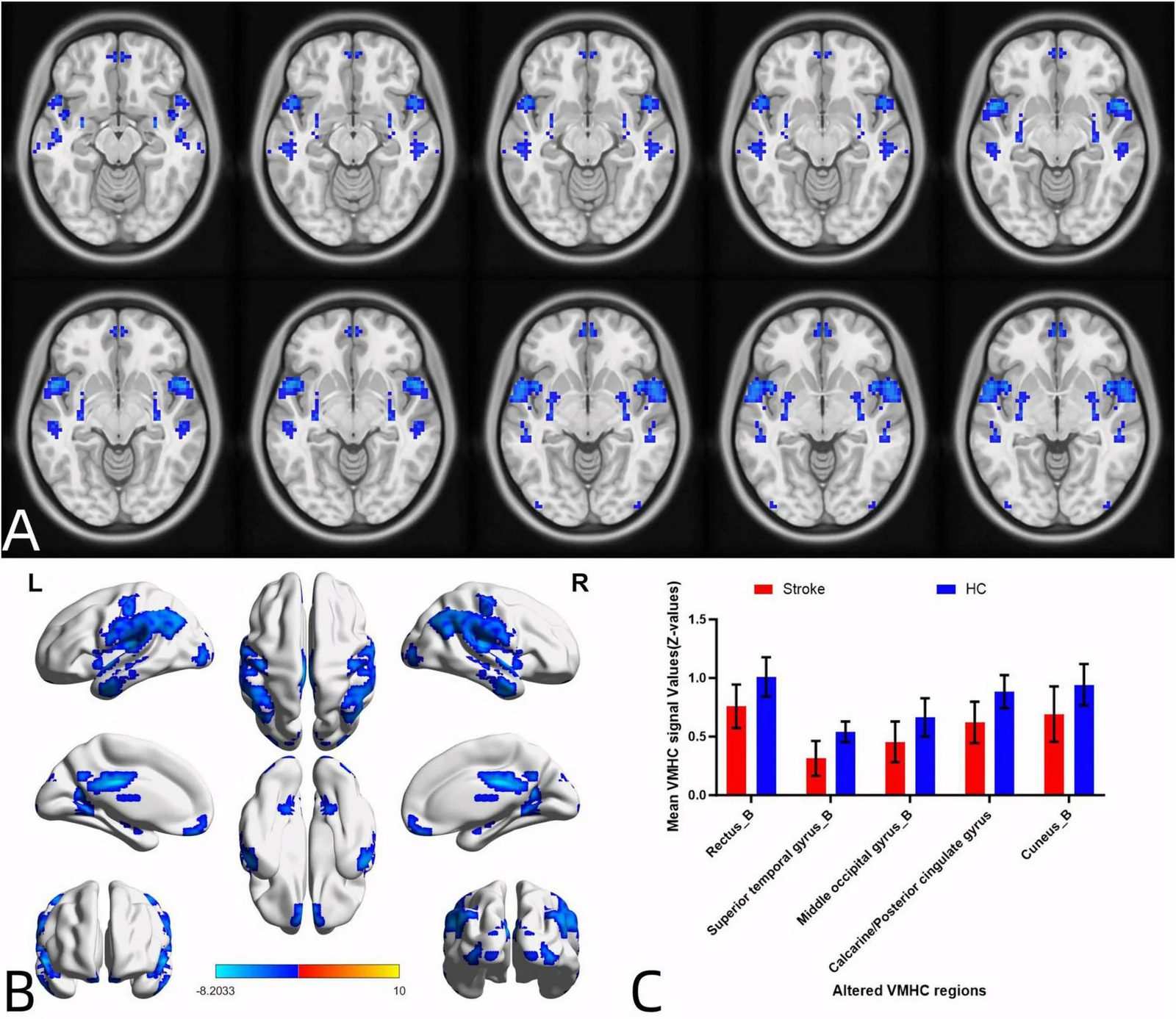
Group differences in VMHC (stroke < control). The differences in VMHC signal values between stroke patients and HC group are shown in panels **(A)** and **(B)**. In panel **(C)**, the mean VMHC changes in the stroke patient group are represented in a histogram. Blue-indigo color indicates decreased VMHC in stroke patients. Significant reductions were observed in REC, STG, MOG, CUN, and CAL.R/PCG.L (voxel level: *p* < 0.001, GRF correction; cluster level: *p* < 0.05). GRF, Gaussian random field; HC, healthy control; L, left; R, right.

One-sample *t*-test results (Figures 2A, B) showed that both groups exhibited typical high interhemispheric VMHC patterns (*p* < 0.05). Compared with controls, stroke patients showed significantly reduced VMHC in multiple brain regions: bilateral rectus gyrus (REC), superior temporal gyrus (STG), middle occipital gyrus (MOG), cuneus (CUN), and right calcarine/left posterior cingulate gyrus (CAL.R/PCG.L) (Figure 3, Table 2; voxel *p* < 0.001, GRF cluster *p* < 0.05).

**TABLE 2:** Brain regions with significantly decreased VMHC in stroke patients (MNI coordinates, cluster size, peak T-score).

Conditions	Brain regions	*x*	*y*	*z*	Cluster size	T-score of peak voxel
Stroke < HC	Rectus gyrus_B	±3	51	−27	78	−4.8946
Stroke < HC	Superior temporal gyrus_B	±54	−3	0	2282	−8.2033
Stroke < HC	Middle occipital gyrus_B	±39	−87	0	132	−5.1886
Stroke < HC	Calcarine/Posterior cingulate gyrus	±6	−24	27	227	−7.6564
Stroke < HC	Cuneus_B	±6	−90	36	63	−5.3167

The statistical values of peak voxels demonstrating significant differences between the Stroke and HC groups are presented. The coordinates (X, Y, Z) correspond to the primary peak locations in the MNI space. The analysis was performed at a voxel level of *P* < 0.001, with GRF correction for multiple comparisons at a cluster level of *P* < 0.05. MNI, Montreal Neurological Institute; B, both; REC, rectus gyrus; STG, superior temporal gyrus; MOG, middle occipital gyrus; CUN, cuneus; CAL.R, right calcarine; PCG.L, left posterior cingulate gyrus.

After controlling for age and sex, mean VMHC across significant regions negatively correlated with NIHSS total score (partial *r* = −0.49, *p* = 0.005). CAL.R/PCG.L VMHC showed the strongest negative correlation (partial *r* = −0.60, *p* < 0.001), as well as with “consciousness level” and “gaze” subscales. STG VMHC negatively correlated with the “language” subscale (partial *r* = −0.45, *p* = 0.012). Regression analysis indicated that CAL.R/PCG.L VMHC (β = −0.41, 95% CI: [−0.62, −0.20], *p* < 0.001) and STG VMHC (β = −0.27, 95% CI: [−0.48, −0.06], *p* = 0.012) together explained 42% of NIHSS variance (adjusted R^2^ = 0.42, F = 9.8, *p* < 0.001).

### Genes related to VMHC differences

PLS regression identified 4 significant latent components explaining 42% of the variance in VMHC *t*-values (cumulative R^2^ = 0.42; Component 1: 18%, Component 2: 12%, Component 3: 7%, Component 4: 5%). A total of 1,198 positively correlated genes and 1,198 negatively correlated genes were identified (*p* < 0.001). These sets were used for subsequent enrichment and PPI analyses.

### Gene enrichment results

**FIGURE 4:**
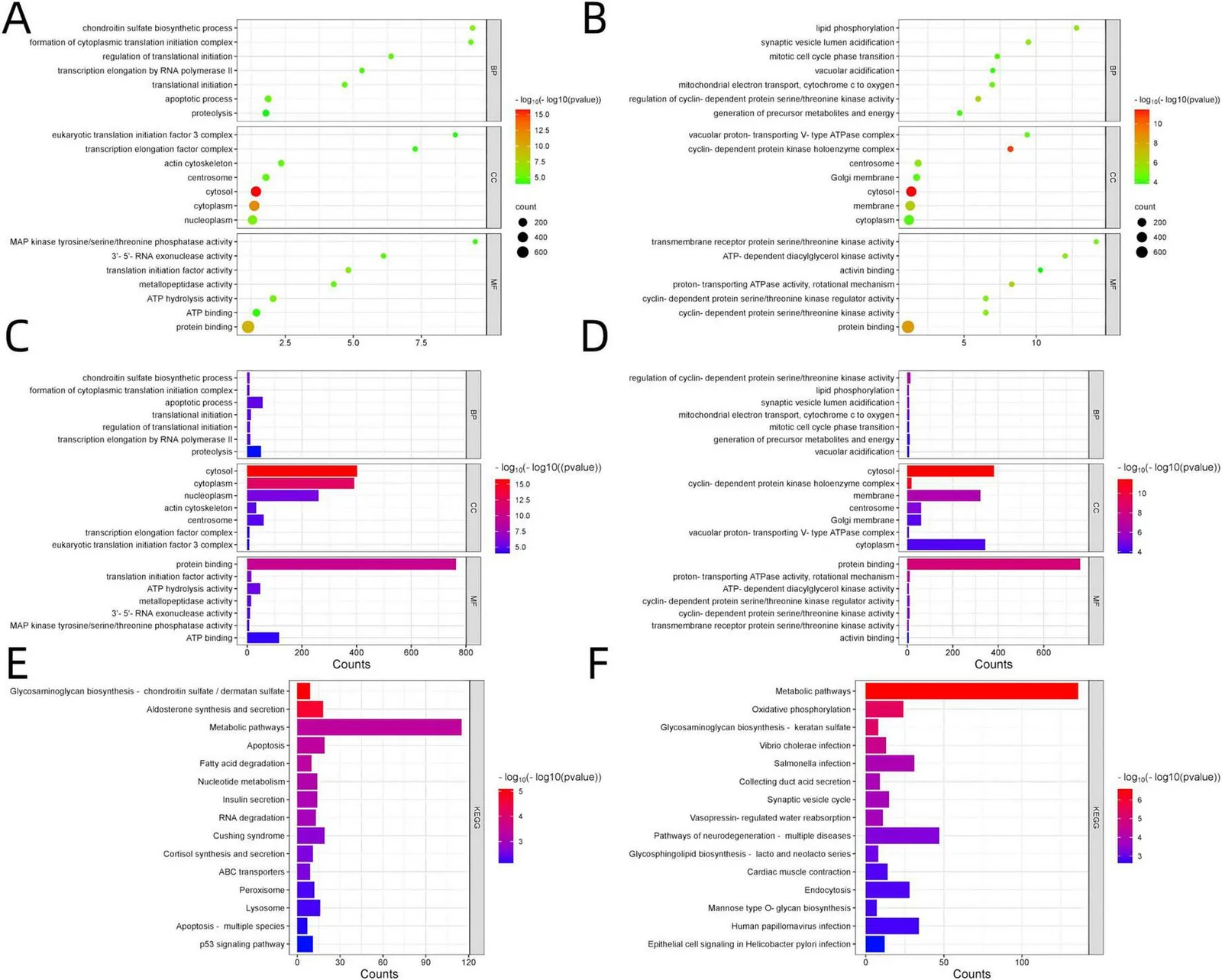
GO and KEGG enrichment analyses. **(A–D)** Gene Ontology terms (BP, CC, MF) for positively **(A, C)** and negatively **(B, D)** correlated gene sets. **(E, F)** KEGG pathway enrichment for positive **(E)** and negative **(F)** sets. Bubble size indicates gene count; color represents FDR-adjusted significance (red: lower p). BP, biological process; CC, cellular component; MF, molecular function; KEGG, Kyoto Encyclopedia of Genes and Genomes.

For the positively correlated gene set (Figures 4A, C, E), GO analysis revealed enriched biological processes including chondroitin sulfate biosynthetic process, cytoplasmic translation initiation complex formation, regulation of translation initiation, transcription elongation by RNA polymerase II, translational initiation, apoptotic process, and proteolysis. Enriched cellular components included the eukaryotic translation initiation factor 3 complex, transcription elongation factor complex, actin cytoskeleton, centrosome, cytosol, cytoplasm, and nucleoplasm. Enriched molecular functions included MAP kinase tyrosine/serine/threonine phosphatase activity, 3′-5′-RNA exonuclease activity, translation initiation factor activity, metallopeptidase activity, ATP hydrolysis, ATP binding, and protein binding. KEGG pathways included glycosaminoglycan biosynthesis (chondroitin sulfate/dermatan sulfate); aldosterone synthesis and secretion; metabolic pathways; apoptosis; and fatty acid degradation (Figure 4E).

For the negatively correlated gene set (Figures 4B, D, F), GO analysis revealed enriched biological processes including lipid phosphorylation, synaptic vesicle lumen acidification, mitotic cell cycle phase transition, vacuolar acidification, mitochondrial electron transport (cytochrome c to oxygen), regulation of cyclin-dependent serine/threonine kinase activity, and synthesis of metabolic intermediates. Cellular components prominently featured vacuolar proton-transporting V-type ATPase, cyclin-dependent kinase holoenzyme, centrosome, Golgi membrane, and cytosol-membrane-cytoplasm networks. Molecular functions included serine/threonine kinase receptor activity, ATP-dependent diacylglycerol kinase activity, proton-transporting ATPase activity, and cyclin-dependent kinase regulator activity. KEGG pathways included metabolic pathways, oxidative phosphorylation, glycosaminoglycan biosynthesis (keratan sulfate), and infectious disease pathways (Vibrio cholerae, Salmonella) (Figure 4F).

### Protein-protein interaction network and hub genes

**FIGURE 5:**
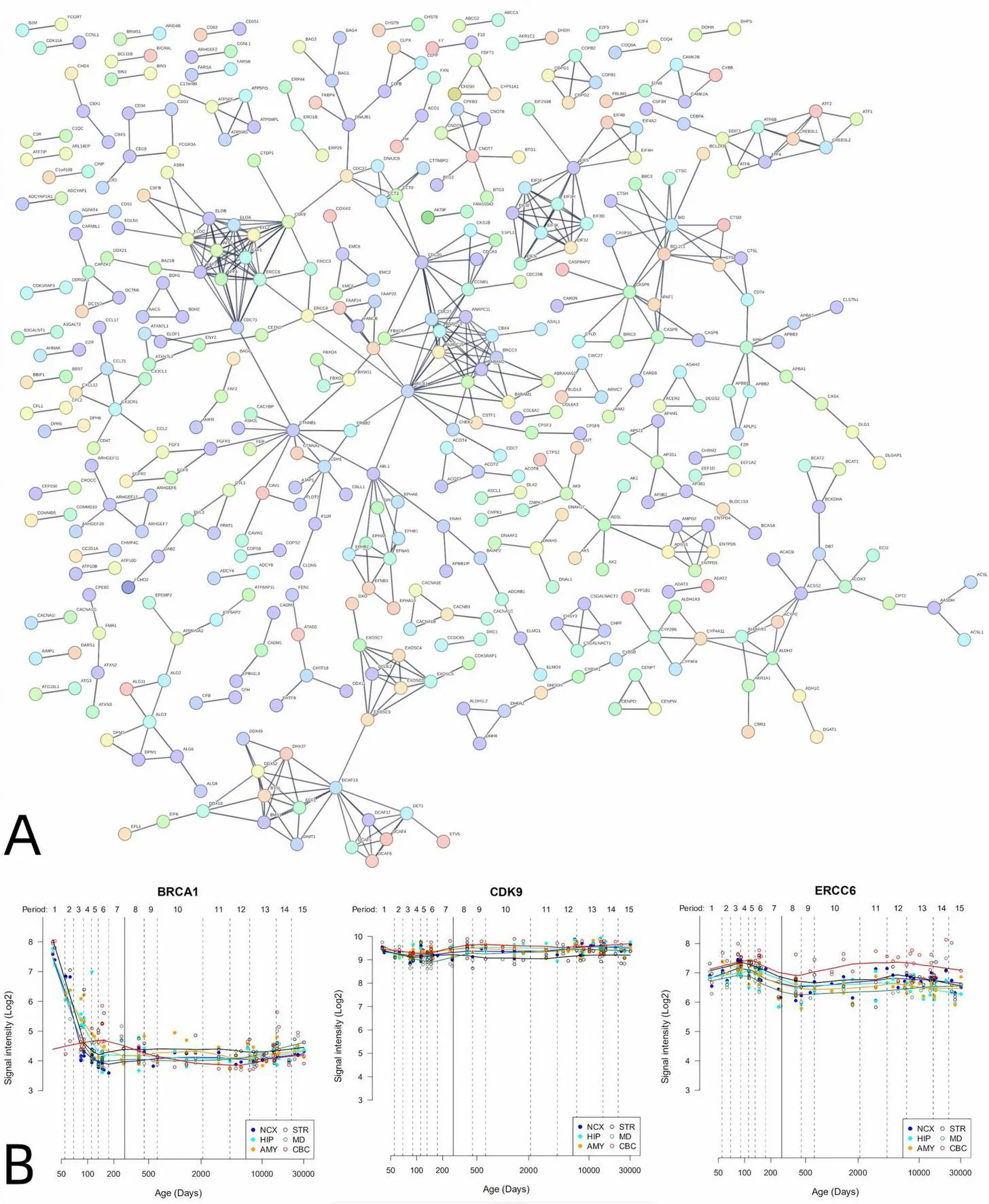
PPI network and hub genes (positive set: BRCA1, CDK9, ERCC6). PPI network **(A)** and spatiotemporal expression **(B)** for positively correlated hub genes (*BRCA1*, *CDK9*, *ERCC6*). Nodes are colored by degree (darker = higher connectivity); edge thickness represents STRING confidence score. In panel **B**, *x*-axis represents developmental stages (prenatal, infancy, childhood, adolescence, adulthood); *y*-axis shows normalized expression levels; shaded areas indicate ± SEM.

**FIGURE 6:**
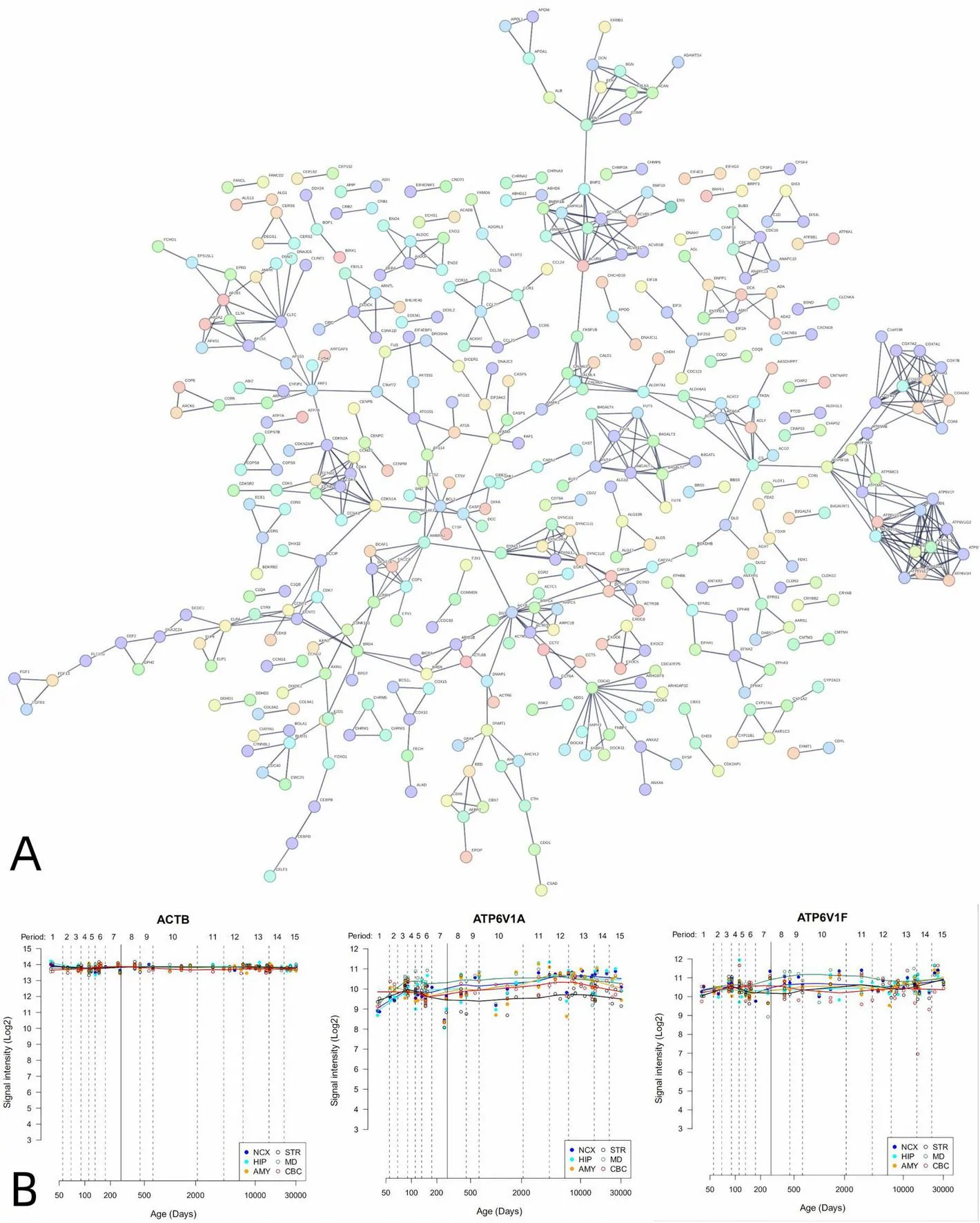
PPI network and hub genes (negative set: ACTB, ATP6V1A, ATP6V1F). PPI network **(A)** and spatiotemporal expression **(B)** for negatively correlated hub genes (*ACTB*, *ATP6V1A*, *ATP6V1F*). Nodes are colored by degree (darker = higher connectivity); edge thickness represents STRING confidence score. In panel **B**, x -axis represents developmental stages (prenatal, infancy, childhood, adolescence, adulthood); *y*-axis shows normalized expression levels; shaded areas indicate ± SEM.

PPI networks were successfully constructed (Figures 5A, 6A). The positively correlated network comprised 1,142 nodes, and the negatively correlated network comprised 1,131 nodes; both were statistically significant (PPI enrichment *p* < 1.0 × 10^−16^). The positive network exhibited an average node degree of 4.2, clustering coefficient of 0.38, and network density of 0.041. The negative network showed an average degree of 3.9, clustering coefficient of 0.35, and density of 0.039. Both networks had significantly higher clustering coefficients than random networks (*p* < 0.001 for both), indicating small-world properties. Hub genes (top 10% node degree) included BRCA1 (BRCA1 DNA repair associated), CDK9 (cyclin dependent kinase 9), and ERCC6 (ERCC excision repair 6) in the positive set, and ACTB (actin beta), ATP6V1A (ATPase H+ transporting V1 subunit A), and ATP6V1F (ATPase H+ transporting V1 subunit F) in the negative set. Spatiotemporal expression analysis showed peak expression in cortical regions known to be vulnerable to ischemic injury, including the middle temporal gyrus (Brodmann area 21), inferior parietal lobule (BA 40), and superior frontal gyrus (BA 8), based on the Human Brain Transcriptome Atlas regional annotation. Vulnerability was defined as regions with high metabolic demand and limited collateral circulation, based on prior stroke vulnerability mapping studies (Figures 5B, 6B). A summary of the principal findings, including altered brain regions, clinical correlations, gene association statistics, and hub genes, is provided in Table 3.

**TABLE 3:** Summary of principal findings.

Domain	Key finding	Statistic
VMHC reductions	STG, MOG, CUN, REC, CAL.R/PCG.L	*p* < 0.05, GRF-corrected
Clinical correlation (partial)	Mean VMHC vs. NIHSS	partial *r* = −0.49, *p* = 0.005
	CAL.R/PCG.L vs. NIHSS	partial *r* = −0.60, *p* < 0.001
	STG vs. language subscale	partial *r* = −0.45, *p* = 0.012
Gene associations	Positively correlated genes	*n* = 1,198
	Negatively correlated genes	*n* = 1,198
PLS model	Latent components (cumulative R^2^)	4 components, R^2^ = 0.42
Hub genes (positive)	*BRCA1, CDK9, ERCC6*	Top 10% node degree
Hub genes (negative)	*ACTB, ATP6V1A, ATP6V1F*	Top 10% node degree

Partial correlations for clinical metrics controlled for age and sex. PLS, partial least squares; PPI, protein-protein interaction.

## Discussion

This study integrates resting-state fMRI and spatial transcriptomic data to investigate the genetic correlates of interhemispheric functional decoupling in ischemic stroke patients. It extends previous multimodal work (Zhang et al., 2024) by linking stroke-related VMHC alterations with specific gene expression patterns. We found reduced VMHC in temporal, occipital, and cingulate regions, and identified 2,396 correlated genes enriched in synaptic function, energy metabolism, and neuroinflammation. PPI analysis highlighted hub genes (*BRCA1*, *CDK9*, *ACTB*, *ATP6V1A/F*) as potential molecular correlates of post-stroke network reorganization. Importantly, all gene-VMHC associations reported here are correlational and do not imply directional causality; they should be interpreted as hypothesis-generating findings.

### Interpretation of VMHC reductions

The observed reduction in STG VMHC is consistent with post-stroke language and working memory deficits (Warrington and Shallice, 1969; Leff et al., 2009). The STG plays a key role in auditory short-term memory and speech comprehension (Mesulam, 1990), and connects to temporo-limbic and neocortical association circuits (Pearlson, 1997). Reduced VMHC in MOG and cuneus may reflect damage to the primary visual cortex (V1), contributing to contralateral hemianopia after occipital stroke (Tharaldsen et al., 2020). The precuneus/posterior cingulate (PCG) is a core node of the default mode network (Buckner et al., 2008); its reduced homotopic connectivity has been associated with amnestic syndromes and spatial cognitive deficits (Valenstein et al., 1987; Burgess et al., 2000). Importantly, all these cortical VMHC reductions occurred in patients with basal ganglia or semiovale infarcts – deep subcortical lesions. This suggests diaschisis: focal subcortical damage can cause remote functional impairment in connected cortical areas via disrupted thalamocortical or striato-cortical pathways (Carrera and Tononi, 2014).

### Genetic pathways underlying VMHC changes

Positively correlated genes highlight extracellular matrix (ECM) remodeling and transcriptional regulation. Chondroitin sulfate proteoglycans (CSPGs) are major ECM components that limit post-stroke plasticity (Pizzorusso et al., 2002). CSPGs regulate excitatory/inhibitory balance by affecting parvalbumin-positive interneurons (Miyata et al., 2012); imbalance is common in stroke and other neurological disorders (Lewis et al., 2012; Verret et al., 2012). RNA polymerase II (RNAPII) dysfunction – enriched in our positive set – is linked to neurodevelopmental disorders with hypotonia and intellectual disability (Haijes et al., 2019), suggesting that transcriptional regulation capacity may influence post-stroke functional outcomes.

Negatively correlated genes strongly implicate mitochondrial energy metabolism and synaptic vesicle acidification. Ischemic stroke depletes ATP, causing mitochondrial Ca^2+^ overload, opening of the mitochondrial permeability transition pore (MPTP), and excessive reactive oxygen species (ROS) production (Jin et al., 2016; Watts et al., 2013). Mitochondria-induced apoptosis via cytochrome c release further disrupts ATP synthesis (Desagher and Martinou, 2000). Our enrichment of “mitochondrial electron transport” and “vacuolar acidification” directly supports this mechanism. Synaptic vesicle acidification, driven by V-ATPase, is essential for neurotransmitter loading; its disruption may impair interhemispheric signaling efficiency.

### Hub genes and potential mechanisms

*BRCA1* has been shown in preclinical studies to be involved in DNA repair, oxidative stress response, and apoptosis (Christou and Kyriacou, 2013). Its expression in neural stem cells is linked to their proliferation (Korhonen et al., 2003). Ischemia-induced oxidative stress depletes neural stem cells (Nakagomi et al., 2009), and *BRCA1* overexpression has been reported to mitigate apoptosis in experimental stroke models (Xu et al., 2019). In our correlational analysis, higher VMHC was associated with higher *BRCA1*-related expression, raising the hypothesis that *BRCA1* may play a compensatory role in preserving interhemispheric connectivity, though this requires direct mechanistic testing.

CDK9 regulates transcription elongation by RNAPII (Cramer, 2019). Xia et al. found that CDK9 upregulates p53 transcription in ischemic neurons, disrupting mitochondrial energy metabolism and aggravating infarction in experimental models (Xia et al., 2024). Our finding that CDK9 expression correlated positively with VMHC suggests a potential association with transcriptional regulatory capacity, though causality cannot be inferred.

*ERCC6* links transcription and DNA repair (Selby and Sancar, 1991); its expression increases after cerebral ischemia in animal models (Ling et al., 1999), and dysfunction causes neurological deterioration with hypomyelination (Shehata et al., 2014). The positive correlation observed in our study is consistent with the hypothesis that these three hubs may represent compensatory transcriptional responses, but this remains speculative.

Among negatively correlated hubs, *ACTB* encodes β-actin, a cytoskeletal protein regulating endothelial nitric oxide synthase (eNOS) and nitric oxide production (Su et al., 2003); actin aggregation increases vascular resistance and stroke risk in preclinical models (Moustafa-Bayoumi et al., 2007). ATP6V1A and ATP6V1F encode subunits of the V-type proton ATPase (V-ATPase), which is essential for lysosomal and synaptic vesicle acidification. Disruption of V-ATPase function has been shown to impair synaptic vesicle filling and neurotransmitter release in experimental systems, while ATP6V1F has additionally been implicated in immune-related pathways (Shen et al., 2023). Our finding that reduced expression of these genes correlates with greater VMHC reduction suggests a potential link to impaired vesicular function and interhemispheric transmission, though causal direction cannot be determined.

### Translational limitations

The hub genes identified in this study – *BRCA1*, *CDK9*, *ERCC6*, *ACTB*, *ATP6V1A*, and *ATP6V1F* – have established roles in diverse biological processes based on preclinical models. However, several caveats must be noted before considering these genes as therapeutic targets. First, our findings are correlational and derived from healthy brain transcriptomics, not from stroke-affected tissue. Second, the direction of association (positive or negative with VMHC) does not imply a beneficial or detrimental effect; it simply reflects co-variation across brain regions. Third, the functional significance of these genes in the human post-stroke brain remains largely unknown. Thus, these findings should be viewed as hypothesis-generating rather than as validated targets for therapeutic intervention.

### Limitations

Several limitations should be acknowledged. First, AHBA data are derived from healthy postmortem brains, not from stroke patients. Thus, our analysis correlates stroke-induced VMHC changes with a healthy transcriptomic baseline, not with dynamic post-ischemic gene expression. This means we identify regions whose constitutive genetic profile is associated with connectivity vulnerability, rather than stroke-induced transcriptional shifts. Future studies using peri-infarct tissue or animal models are needed. Second, the cross-dataset design (imaging in patients, transcriptomics in healthy donors) introduces noise, though phylogenetic conservation of brain-wide gene expression patterns supports population-level consistency (Hawrylycz et al., 2012). Third, the use of uncorrected whole-brain *t*-values as the PLS response variable increases sensitivity to diffuse signals but may inflate false positives; we disclosed this choice and note that future region-restricted analyses are warranted. Fourth, limited right-hemisphere expression data in the AHBA forced a focus on left hemisphere samples, introducing potential lateralization bias. Since interhemispheric functional homotopy is inherently a bilateral phenomenon, this asymmetry may have influenced our gene-VMHC associations. Future studies incorporating bilateral transcriptomic data are needed to confirm the generalizability of our findings. Fifth, residual head motion effects may persist despite correction. Sixth, while we have provided detailed characterization of lesion side (left/right hemisphere) and volume, we did not perform subgroup analyses based on lesion volume or specific vascular territories due to sample size constraints (*n* = 50). Future studies with larger cohorts are needed to systematically evaluate the impact of lesion volume and vascular distribution on post-stroke VMHC alterations. Importantly, all lesions in our cohort were confined to the basal ganglia and semiovale center, which share a common vascular supply, thereby reducing the extent of inter-individual variability in vascular territory involvement. Seventh, the cross-sectional design captures a single time point in the acute phase. Observed VMHC abnormalities may represent transient phenomena rather than stable features of network reorganization. Longitudinal imaging data are necessary to determine whether the identified transcriptomic signatures are associated with recovery trajectories or chronic reorganization processes. Despite these limitations, cross-validation and permutation testing supported model stability.

## Conclusion

This multimodal integration study identifies polygenic correlates associated with abnormal interhemispheric functional homotopic connectivity (VMHC) in stroke patients. Reduced VMHC was observed in temporal, occipital, and cingulate regions. A total of 2,396 genes correlated with VMHC alterations, with enrichments in synaptic vesicle trafficking, mitochondrial energy metabolism, and neuroinflammation. PPI network analysis highlighted hub genes including *BRCA1*, *CDK9*, *ACTB*, and *ATP6V1A/F* as potential molecular correlates. These findings provide a framework linking macroscale connectivity disruption to specific molecular pathways, offering testable hypotheses for future mechanistic and therapeutic studies in stroke recovery. All gene-imaging associations are correlational and require independent validation.

## Data Availability

The datasets presented in this study can be found in online repositories. The names of the repository/repositories and accession number(s) can be found below: Data are available at Figshare (https://doi.org/10.6084/m9.figshare.29646134). AHBA data from https://human.brain-map.org. Abagen toolbox at https://abagen.readthedocs.io.

## Footnotes

1
https://abagen.readthedocs.io/

2
http://www.bioinformatics.com.cn/

3
http://hbatlas.org/
